# Comprehensive estimation of input signals and dynamics in biochemical reaction networks

**DOI:** 10.1093/bioinformatics/bts393

**Published:** 2012-09-03

**Authors:** M. Schelker, A. Raue, J. Timmer, C. Kreutz

**Affiliations:** ^1^Institute for Physics; ^2^Freiburg Center for Systems Biology (ZBSA), University of Freiburg, D-79104 Freiburg, Germany; ^3^Freiburg Institute for Advanced Studies (FRIAS); ^4^BIOSS Centre for Biological Signalling Studies, University of Freiburg, Germany; ^5^Department of Clinical and Experimental Medicine, Linköping University, SE-581 83 Linköping, Sweden

## Abstract

**Motivation:** Cellular information processing can be described mathematically using differential equations. Often, external stimulation of cells by compounds such as drugs or hormones leading to activation has to be considered. Mathematically, the stimulus is represented by a time-dependent input function.

Parameters such as rate constants of the molecular interactions are often unknown and need to be estimated from experimental data, e.g. by *maximum likelihood estimation*. For this purpose, the input function has to be defined for all times of the integration interval. This is usually achieved by approximating the input by interpolation or smoothing of the measured data. This procedure is suboptimal since the input uncertainties are not considered in the estimation process which often leads to overoptimistic confidence intervals of the inferred parameters and the model dynamics.

**Results:** This article presents a new approach which includes the input estimation into the estimation process of the dynamical model parameters by minimizing an objective function containing all parameters simultaneously. We applied this comprehensive approach to an illustrative model with simulated data and compared it to alternative methods. Statistical analyses revealed that our method improves the prediction of the model dynamics and the confidence intervals leading to a proper coverage of the confidence intervals of the dynamic parameters. The method was applied to the *JAK-STAT signaling pathway*.

**Availability:** MATLAB code is available on the authors' website http://www.fdmold.uni-freiburg.de/~schelker/.

**Contact:** max.schelker@fdm.uni-freiburg.de

**Supplementary Information:** Additional information is available at *Bioinformatics Online*.

## 1 INTRODUCTION

Mathematical modeling of biological systems has become a widely used approach to better understand the system behavior as a whole rather than observing isolated parts (Kitano, 2002). The rapid development in quantitative molecular biology (Cox and Mann, 2011) enables to calibrate mathematical models to experimental data and therefore generating model predictions. For parametric models, such as ordinary differential equation (ODE) models, calibration can be performed e.g. by *maximum likelihood estimation* (MLE). Uncertainties of the measurements generated by biological variability or technical limitations have to be considered in the calibration process as they propagate to the parameter estimates. An accurate method for calculating confidence intervals is given by the *profile likelihood approach* (Raue *et al.*, 2009).

One unresolved issue in data-based modeling is the insufficient consideration of input measurement uncertainties in the parameter estimation process. In this article, we solve this problem with a new approach. The method presented in the following includes the input estimation into the estimation process of the dynamical model parameters by minimizing an objective function containing all parameters simultaneously. An illustrative model with known parameter values and noise distributions reveals that our method is able to (i) increase the precision of the parameter estimates and (ii) correct the coverage of the likelihood-based confidence intervals.

Furthermore, we applied the method to a model of the JAK-STAT signaling pathway introduced by Swameye *et al.* (2003) where quantitative biological data are available, leading to different estimated dynamics of the model trajectories compared to the standard method.

## 2 METHODS

### 2.1 Models of biochemical reaction networks

A mathematical model ℳ of a biochemical reaction network is given by a set of coupled ODEs
(1)
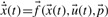

(2)
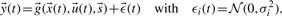

where *x_i_* (*t*) are the *internal states* of the system, *u_i_* (*t*) are the time dependent *external stimuli*, also called *input functions* and *y_i_* (*t*) are the noisy observables of the system with a noise level σ*_i_* .

The set of parameters that defines ℳ uniquely contains *dynamic parameters* 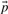, i.e. for instance rate constants, *scaling* and *offset* parameters 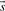 as well as the initial concentrations 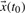

(3)
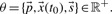

All analyses have been performed on a logarithmic scale.

### 2.2 Parameter estimation

We performed MLE which is equivalent to the weighted least squares method for Gaussian noise. Thus, an *objective function*
(4)
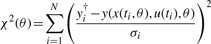

is minimized and
(5)
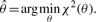

is the maximum likelihood estimator where *y_i_*^†^ denotes the measured data and *y*(*x*(*t_i_*,*θ*),*u*(*t_i_*), *θ*) represents the model output. In order not to rely on the mere point estimate, likelihood profiles will be computed as discussed in more detail in the Supplementary Information.

### 2.3 Existing methods

For integration of the ODEs, a continuous representation of a discretely measured input is required. In the past, time-dependent input functions *u*(*t*) have been represented by constant input functions Bachmann *et al.* (2011), linear interpolations of the dataset (Quach *et al.*, 2007; Swameye *et al.*, 2003), cubic interpolation splines or by cubic smoothing splines (Maiwald and Timmer, 2008; Raue *et al.*, 2009).

If the functional representation of the input data is estimated separately from the dynamical model parameters, the deviations from the true underlying dynamics of the input are not considered in the estimation of the model parameters. This can result on the one hand in biased estimates of the dynamic parameters and on the other hand, even more likely, in underestimated confidence intervals as the input uncertainties have not been taken into account. This problem shall be addressed in the following by defining a suitable input parametrization that enables to estimate input and dynamical parameters comprehensively.

### 2.4 Splines

A spline is a piecewise polynomial that interpolates a given set of data points *y*_1_^†^,...,*y_N_*^†^ at time points *t*_1_,...,*t_N_* in a smooth and continuous manner. The *y*-values to be interpolated are called *control points* and the corresponding *t*-values *knots*. *Boundary conditions* determine either the slope or the curvature of the spline at the first and the last data point. Here, *cubic splines* with *natural boundary conditions*, i.e. the curvature at both end points is set to zero, are utilized.

A *cubic interpolation spline* can be described either by a set of knots and the corresponding four coefficients for each polynomial piece or by a linear combination of a set of basis functions in the *basis spline* (B-spline) notation. For a knot sequence *t*_1_,...,*t_N_* and its corresponding control points *v*_1_,..., *v*_N_, the B-spline is given by (de Boor, 1978)
(6)
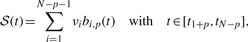

where *N* is the number of data points and *p* is the *degree* of the basis functions *b_i,p_*(*t*), thus, in case of cubic polynomials, *p* = 3. The closed-form expression of the basis functions is given in the Supplementary Information.

Another type of spline, that is frequently used, is the cubic smoothing spline. A smoothing spline
(7)
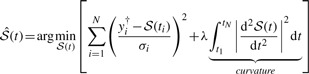

can, depending on its respective measurement uncertainties σ*_i_*, deviate from the data points *y_i_*^†^ in order to form a smoother curve. Therefore, an objective function is to be minimized, containing both the deviation from the data and the curvature, i.e. the second term in (7). The so-called smoothing or penalization parameter λ∈ [0,∞) controls the trade-off between interpolating the noisy data and obtaining a smooth function. As a consequence, λ = 0 results in an interpolation spline, whereas λ → ∞ corresponds to a linear fit of the input data.

Interpolation splines often lead to strongly oscillating input function estimates. Therefore, smoothing splines usually lead to more realistic outcomes if the smoothing parameter λ can be chosen reasonably.

A smoothing spline can be unambiguously represented either by the data points *y*_1_^†^,...,*y*_*N*_^†^, the knot sequence *t*_1_,...,*t_N_* and the smoothing parameter λ or by the knots *t*_1_,...,*t_N_* and the corresponding control points *v*_1_,...,*v*_*N*_, that are the obtained by evaluation of Equation 7, i.e. *v_i_*=*Ŝ*(*t_i_*). Therefore, for the approach presented in the following, the control points *v*_1_,...,*v_N_* are used as input parametrization even though the spline is smoothed by penalizing the curvature.

### 2.5 Assessment criteria

The performance of the approach is assessed by the accuracy
(8)
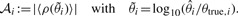

and the *precision*
(9)
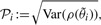

i.e. the standard deviation of the distribution of the parameter estimates, if many independent estimations are performed over simulated data for known true parameter values.

Moreover, the deviation from the true trajectory is quantified by the score
(10)
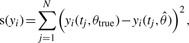

where *t_j_* ∈ [*t*_0_,*t*_end_] is densely sampled at 500 equidistant time points.

The coverage ratio, i.e. the probability that the true parameters are inside the confidence intervals, is calculated using the likelihood-based confidence intervals for many noise realization as described in more detail in the Supplementary Information. This value can be compared to the desired coverage, i.e. the corresponding α level.

### 2.6 Approach

The new approach aims to include the estimation of the input function into the maximum likelihood estimation of the model parameters. Therefore, a parametrization for the input measurements is needed.

A cubic spline is uniquely defined by the set of data points it is fitted to. This is best visible in the B-spline notation introduced in Equation 6. Thus, the input can be parametrized using the *control points v*_1_,..., *v_N_* of the spline as parameters.

The optimal set of parameters is then obtained by minimizing an objective function
(11)
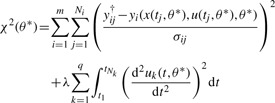

that contains both input and downstream parameters as well as a term that penalizes the curvature of the input functions. The smoothing parameter λ is selected based on χ^2^-statistic as described in the Supplementary Information. Note that the first term in Equation 11 accounts for the deviation of the model *y_i_*(*t_j_*) from the data *y_ij_*^†^ for all observables including the input.

The extended parameter set to be optimized is
(12)
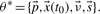

In the following, θ^*^→ *θ* will be used to keep the notation as simple as possible.

Input measurements are usually concentrations or relative measurements like receptor activation and therefore strictly positive. A spline can become negative between the positive measurement points leading to reversed reaction fluxes and incorrect model behavior. To avoid negativity of the spline function, the input is transformed to logarithmic scale.

## 3 APPLICATIONS

In the following, the novel approach, which will also be called *comprehensive approach*, is compared to the *standard approach*, where the input is estimated from the measurement data *before* the model parameters are calibrated. In Section 3.1 the smoothing parameter λ was set to zero for both the standard and the comprehensive approach. By contrast, for both approaches in Section 3.2, the smoothing parameter was chosen optimally to the measured data based on χ^2^-statistics as described in the Supplementary Information.

### 3.1 An illustrative model

**Fig. 1.**
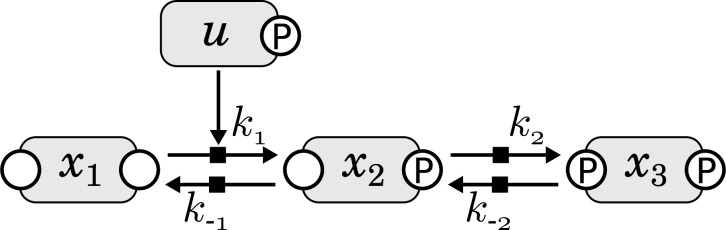
Schematic representation of the illustrative model. The forward reaction from X_1_ to X_2_ is catalyzed by the input *u*. This reaction is reversible with a rate constant *k*_−1_. Furthermore, X_2_ reacts to X_3_ with rate constants *k*_2_ and *k*_−2_

The first application of the new approach is a toy model with three dynamic states X_1_,X_2_ and X_3_ and one input variable *u*. As depicted in Figure 1, the model consists of two reversible reactions where the first forward reaction (X_1_ → X_2_) is catalyzed by a time-dependent input function *u*(*t*). The second forward reaction (X_2_ → X_3_) as well as both reverse reactions (X_3_ → X_2_ and X_2_ → X_1_) are considered to be first-order mass action kinetics and do not depend on the input directly.

The ODE system describing the dynamics of the model is given in the Supplementary Information. The observables are chosen to be *y_i_* = log_10_(*x_i_*) for *i* = 1,2,3 and *y*_4_ = log_10_(*u*) as for protein measurement techniques log-normally distributed measurement uncertainties are assumed (Kreutz *et al.*, 2007).

For the simulation, *N* = 12 time points between *t*_0_ = 0 and *t*_end_ = 50 were chosen with an equidistant sampling. The kinetic rate constants were set to *k*_1_ = 0.01, *k*_−1_ = 1, *k*_2_ = 0.5 and *k*_−2_ = 0.1. The initial concentrations were chosen close to the steady states, i.e. *x*_1_(0) = 30, *x*_2_(0) = 20 and *x*_3_(0) = 50, as this maximizes the impact of the input on the variables. For the underlying true input dynamics, a Gaussian function
(13)
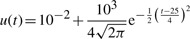

was chosen which has its maximum *u*(*t*_max_) ≈ 100 at *t*_max_ = 25.

In order to evaluate the assessment criteria, *M* = 2000 noise realizations were simulated with a noise level σ_X_ = 0.1 for the compounds X*_i_* and σ*_u_* = 0.3 for the input *u*(*t*). A comparison of several different noise levels is given in the Supplementary Information. For each noise realization, the parameters were estimated as described in Section algorithm in the Supplementary Information.

**Fig. 2.**
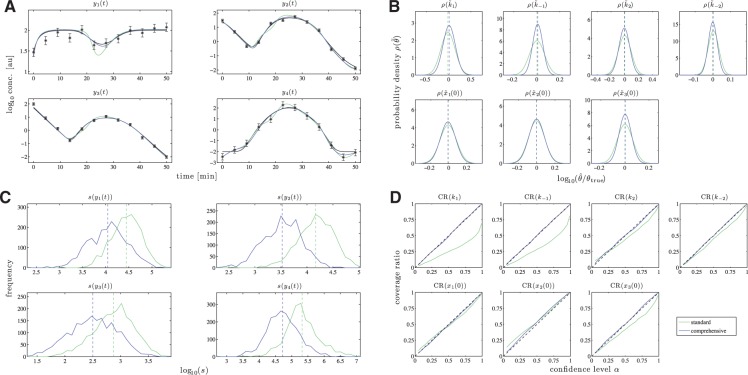
(**A**) Logarithmic plot of model trajectories and simulated data. (**B**) Probability densities for the estimates of the model parameters. (**C**) Score distributions. (**D**) The coverage ratio is plotted against the confidence level to check the size of the confidence intervals. The dashed line indicates identity. In all figures, the green solid line represents the trajectory of the standard approach and the blue one of the comprehensive approach. The black lines indicate the true dynamics

In Panel A of Figure 2, the model trajectories are plotted for one noise realization. The identifiability of the model parameters was analyzed by the profile likelihood approach as described in the Supplementary Information. The likelihood-based confidence intervals were calculated for multiple confidence levels
(14)
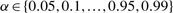

to investigate the coverage.

#### 3.1.1 Evaluation of the assessment criteria

The performance of the novel approach was challenged by the three assessment criteria introduced in Section 2.5. In Panel B of Figure 2, the parameter estimate distributions are shown. In terms of accuracy, both approaches perform similarly well as the dashed lines indicating the mean values are not distinguishable. The precision, by contrast, is increased by a factor of 1.04 up to 1.48 when the novel method is applied. This can also be seen in the plot where all parameter distributions in blue are narrower than the green ones. The values of 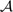 and 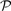 as well as the ratio of these values for both approaches are given in the Supplementary Information.

Panel C of Figure 2 shows that for all four observables, the deviation from the estimated to the true trajectory is significantly smaller in case of the *comprehensive approach*.

In Panel D of Figure 2, the coverage ratio is plotted versus the confidence level for both approaches. In case of the standard approach, the measurement uncertainties of the input are not taken into account. As a consequence, the confidence intervals, at least for some of the parameters, are too small. This results in a coverage ratio that is smaller than the corresponding confidence interval as indicated by the deviation of the green solid line from the black dashed line in Panel D of Figure 2. Underestimating the size of a confidence interval can lead to false-positive rejections of specific values for the respective parameter and therefore represents a serious issue. For the comprehensive approach by contrast, the coverage ratio almost equals the confidence level for all seven model parameters. According to this, the uncertainty of the input measurements is appropriately translated into parameter confidence intervals.

Apparently, the first two rate constants *k*_1_ and *k*_−1_ are affected the most by the choice of the estimation approach. This results from the model structure as the fluxes of the first reaction 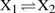 depend directly on the input. Consistently, the coverage of the rate constants *k*_2_ and *k*_−2_ of the second reaction 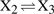 are barely influenced by the input and its uncertainties.

### 3.2 The JAK-STAT signaling pathway

**Fig. 3.**
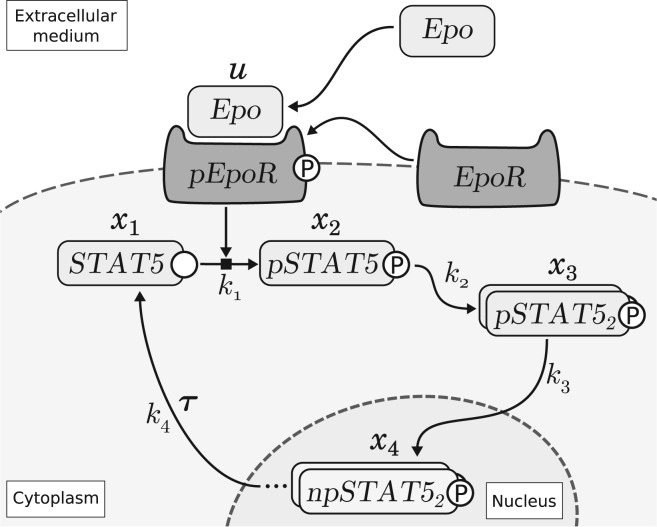
Schematic representation of the JAK-STAT signaling pathway. The phosphorylated Erythropoietin receptor (pEpoR) acts as input for the signaling cascade and induces phosphorylation of STAT5. Phosphorylated STAT5 (pSTAT5) then dimerizes (pSTAT5_2_) and translocates to the nucleus (npSTAT5_2_). There it can act as transcription factor and thereby induce protein synthesis. Furthermore, with some delay τ, the pSTAT5 dimers dissociate to unphosphorylated STAT5 monomers and translocate back to the cytoplasm. (Figure adapted from Swameye *et al.* (2003))

For the second application of the novel approach, a data-based model of JAK2-STAT5 signaling pathway (Swameye *et al.*, 2003) was investigated. In this model the downstream system depends on a measured input. The model structure is depicted in Figure 3. The ODEs describing the dynamics of the model are given in the Supplementary Information.

Swameye *et al.* (2003) performed quantitative immunoblotting in order to investigate the Epo-induced activation of the JAK2-STAT5 signaling pathway. Due to experimental limitations, only sums of several species were measurable leading to the following observables:

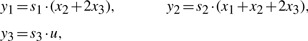

where *y*_1_ is the total amount of phosphorylated STAT5 in the cytoplasm, *y*_2_ the total amount i.e. unphosphorylated and phosphorylated STAT5 in the cytoplasm and *y*_3_ is the measurement of the input function i.e. the amount of the Epo-induced phosphorylation of EpoR. Furthermore, the immunoblotting method could only provide relative protein concentrations. Therefore, scaling parameters *s*_1_ to *s*_3_ are required and the observables are given in arbitrary units (au).

In order to calibrate the model to experimental data, a set of initial conditions for all parameters is needed. First, the initial concentrations of all phosphorylated model species were set to zero:

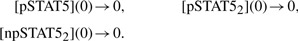


Furthermore, in order to resolve structural identifiability issues, the initial value of STAT5 and the scaling parameter of the input were set to one:

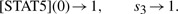

Thereby, the forward rate constant *k*_1_ is expressed in units of the input pEpoR and the total amount of STAT5, which is conserved in this system, is set to one. In a second step, a set of initial guesses for the remaining parameters was selected using Latin hypercube sampling (LHS). Subsequently, the parameters were estimated by maximum likelihood estimation.

**Fig. 4.**
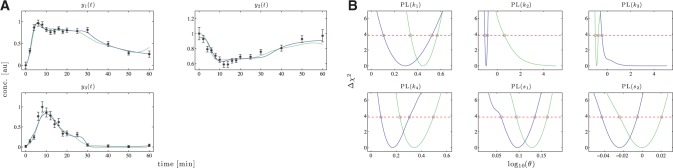
(**A**) Plot of model trajectories and experimental data. (**B**) Likelihood profiles for the model parameters. The red dashed line indicates the confidence threshold for *α* = 0.95. The green solid line represents the likelihood profile calculated with the standard approach and the blue one with the comprehensive approach

**Fig. 5.**
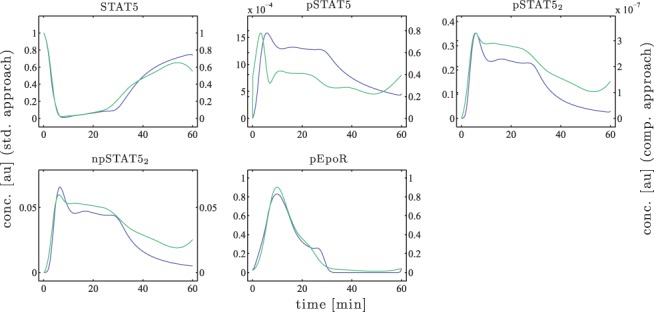
The green solid line represents the trajectory calculated with the standard approach and the blue one with the comprehensive approach. Note the different scaling of the left (standard approach) and the right (comprehensive approach) *y*-axis for pSTAT5 and pSTAT5_2_

Panel A of Figure 4 shows the resulting model trajectories and the corresponding time course data. The model variables, which correspond to the optimal set of parameters for each approach, are depicted in Figure 5. For the model species STAT5, npSTAT5_2_ and for the input pEpoR, the trajectories differ only sightly. However, for pSTAT5 and pSTAT5_2_, a completely different scale is observed for both approaches. This results from parameter values of *k*_2_ and *k*_3_ that differ strongly which will be discussed later on.

The identifiability of the model parameters was analyzed by the profile likelihood approach as described in the Supplementary Information. The resulting likelihood profiles are shown in Panel B of Figure 4. The corresponding values of the parameter estimates and their confidence intervals are given in Table 1.

**Table 1.** Parameter estimates and likelihood-based 95% confidence intervals for both approaches

Parameter [units]	Standard approach			Comprehensive approach		
	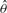	conf_lb_	conf_ub_	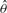	conf_lb_	conf_ub_
*k*_1_ [min^−1^ ·nM^−1^ *s*_3_^−1^]	2.79	2.18	3.70	1.95	1.27	3.33
*k*_2_ [min^−1^ nM^−1^]	1.00×10^5^	4.04	∞	0.11	0.08	0.14
*k*_3_ [min^−1^]	0.12	0.09	0.17	9.84× 10^4^	0.34	∞
*k*_4_ [min^−1^]	2.19	1.69	3.12	1.49	1.20	2.01
*s*_1_ [nM^−1^]	1.34	1.24	1.46	1.25	1.15	1.36
*s*_2_ [nM^−1^]	1.00	0.95	1.05	0.95	0.91	0.99

The parameter estimates of the rate constants *k*_1_ and *k*_4_ can be directly compared to results of Raue *et al.* (2009). However, the point estimates obtained by the standard approach do not exactly match the previously obtained values. This is due to the choice of the input representation: in Raue *et al.* (2009), a smoothing spline of the input data was used without considering the input uncertainties for the weighting of the data points and for the determination of the smoothing parameter. In this article by contrast, the input uncertainties have been estimated from several datasets as described in the Supplementary Information. This allows for a weighting of the data according to the first term in Equation (7) and for a data-based selection of the smoothing parameter λ. In Raue *et al.* (2009), the initial concentration of STAT5 (*x*_1_(0)) and the scaling parameters *s*_1_ and *s*_2_ were structurally non-identifiable. In our article, the parameter value of [STAT5](0) was set to one leading to identifiable scaling parameters having different physical units. Therefore, these parameter values are not comparable with previous results. The results for the rate constants *k*_2_ and *k*_3_ are discussed later on in this article.

#### 3.2.1 Comparison of the results

Here, the results of the standard approach and the comprehensive approach shall be discussed and compared. This analysis requires a different strategy than in Section 3.1 as the underlying truth is not know for this system.

At first, it can be observed that the qualitative behavior of the fits in Panel A of Figure 4 differs between both approaches. The input observable *y*_3_, for instance, has a small plateau at about 25 min in case of the comprehensively estimated input. This is due to downstream information, as in the total concentration of pSTAT5, i.e. the observable *y*_1_, this plateau is also present with some delay. Furthermore, for the standard approach, the model trajectories of *y*_1_ and *y*_2_ deviate significantly from the data at the last measurement time point, i.e. *t* = 60 min. This is due to the input measurement which has a higher value at the last time point (*t* = 60 min) than for the proceeding measurement time points (*t* = 40,50 min). In contrast, in case of the comprehensive approach, the experimental data including the uncertainties of all three observables were considered, leading to model trajectories that better reflect the measured data.

One, at first glance, confusing outcome of the model calibration is the difference in the parameter estimates for *k*_2_ and *k*_3_ as depicted by the likelihood profiles in Panel B of Figure 4. These two parameters show a completely opposed behavior: for the standard approach, the process of dimerization, which is described by *k*_2_, is faster than estimated by the comprehensive approach. In contrast, *k*_3_, which describes the translocation of the pSTAT5 dimers to the nucleus, is smaller for the standard approach than the estimate resulting from the comprehensive approach. As a consequence, the trajectories of the dynamic variables pSTAT5 and pSTAT5_2_ show a different scaling for both estimation approaches. Therefore, in Figure 5, the concentrations obtained by the standard approach are indicated by the *y*-axis on the left and those of the comprehensive approach by the right *y*-axis.

This opposing behavior of *k*_2_ and *k*_3_ was also observed by Raue *et al.* (2009), who investigated the variability of the trajectories when plotting all parameter sets along the likelihood profile that are within the point-wise 95% confidence interval. This results from the fact that only the sum of pSTAT5 and pSTAT5_2_ is measured but not their individual contribution.

**Fig. 6.**
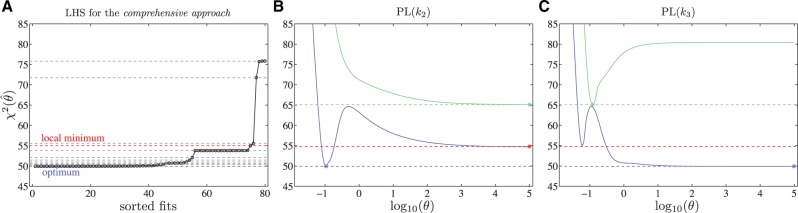
(**A**) Analysis of local minima for the comprehensive approach. Several local minima, indicated by dashed lines, have been identified by LHS. **B** and **C**: Likelihood profiles for *k*_2_ and *k*_3_. The green solid line represents the likelihood profile calculated with the standard approach and the blue one represents the profile around the optimum for the comprehensive approach. The red dashed line and the red asterisks denote a selected local minimum for the comprehensive approach with the switched parameter configuration

Here, the switching from faster dimerization and slower translocation to the opposed configuration can be explained in terms of the χ^2^-landscape. For the comprehensive approach, a local minimum can be identified by LHS, which corresponds to the situation of the standard approach as depicted in Panels A and B of Figure 6. Although both minima are in sufficient agreement with the data because χ^2^ differs by much less than the number of fitted parameters, the optimal point estimates change. The parameter values of the minima are given in the Supplementary Information. However, by optimizing the input in the comprehensive approach, the χ^2^-landscape changes and another minimum with the switched parameter configuration becomes optimal.

The selection of a different local minimum in case of the comprehensive approach also leads to the counterintuitive observation that the confidence intervals for the parameters *k*_4_, *s*_1_ and *s*_2_ are larger for the standard approach than for the novel method.

Hence, the result of the comprehensive approach reveals that an unambiguous estimation of both rates is not feasible based on the measured data. Therefore, additional experiments would be required in order to clarify which parameter configuration corresponds to the real biological situation.

## 4 CONCLUSIONS

It has been shown that the presented approach for estimating model parameters and input comprehensively provides several advantages. For a toy model with simulated data, the three assessment criteria defined in Section 2.5 have been evaluated for different noise level combinations of input and downstream observables. The analysis revealed that the novel approach outperforms the standard method in terms of (i) precision, (ii) deviation from the truth and (iii) coverage of the confidence intervals. The accuracy of the parameter estimates does not differ significantly for both approaches. The performance is best when the measurement uncertainties of the downstream observables are smaller than those of the input measurements.

By applying the new method to a model of the JAK-STAT signaling pathway, it could be shown that also for realistic experimental studies, one benefits from the comprehensive input estimation. The optimal model trajectories indicate that downstream information can be used in order to improve the fit of the input and vice versa.

*Funding*: This work was supported by the German Federal Ministry of Education and Research [Virtual Liver (Grant No. 0315766), LungSys II (Grant No. 0316042G)], the Initiative and Networking Fund of the Helmholtz Association within the Helmholtz Alliance on Systems Biology (SBCancer DKFZ I.2, V.2 and CoReNe HMGU), and the Excellence Initiative of the German Federal and State Governments (EXC 294).

*Conflict of Interest*: none declared.
